# The Origin and Composition of Korean Ethnicity Analyzed by Ancient and Present-Day Genome Sequences

**DOI:** 10.1093/gbe/evaa062

**Published:** 2020-03-27

**Authors:** Jungeun Kim, Sungwon Jeon, Jae-Pil Choi, Asta Blazyte, Yeonsu Jeon, Jong-Il Kim, Jun Ohashi, Katsushi Tokunaga, Sumio Sugano, Suthat Fucharoen, Fahd Al-Mulla, Jong Bhak

**Affiliations:** e1 Personal Genomics Institute (PGI), Genome Research Foundation, Osong, Republic of Korea; e2 Korean Genomics Center (KOGIC), Ulsan National Institute of Science and Technology (UNIST), Ulsan, Republic of Korea; e3 Department of Biomedical Engineering, School of Life Sciences, Ulsan National Institute of Science and Technology (UNIST), Ulsan, Republic of Korea; e4 Department of Archaeology and Art History, Seoul National University, Republic of Korea; e5 Department of Biological Sciences, Graduate School of Medicine, The University of Tokyo, Japan; e6 Department of Human Genetics, Graduate School of Medicine, The University of Tokyo, Japan; e7 Department of Medical Genome Sciences, Graduate School of Frontier Sciences, The University of Tokyo, Japan; e8 Thalassemia Research Center, Institute of Molecular Biosciences, Mahidol University, Nakorn Pathom, Thailand; e9 Center of Genomic Medicine, Kuwait University, Kuwait; e10 Clinomics Inc, Ulsan, Republic of Korea

**Keywords:** Korean origin, Korean migration, population study, paleogenomics, variome, KoVariome

## Abstract

Koreans are thought to be an ethnic group of admixed northern and southern subgroups. However, the exact genetic origins of these two remain unclear. In addition, the past admixture is presumed to have taken place on the Korean peninsula, but there is no genomic scale analysis exploring the origin, composition, admixture, or the past migration of Koreans. Here, 88 Korean genomes compared with 91 other present-day populations showed two major genetic components of East Siberia and Southeast Asia. Additional paleogenomic analysis with 115 ancient genomes from Pleistocene hunter-gatherers to Iron Age farmers showed a gradual admixture of Tianyuan (40 ka) and Devil’s gate (8 ka) ancestries throughout East Asia and East Siberia up until the Neolithic era. Afterward, the current genetic foundation of Koreans may have been established through a rapid admixture with ancient Southern Chinese populations associated with Iron Age Cambodians. We speculate that this admixing trend initially occurred mostly outside the Korean peninsula followed by continuous spread and localization in Korea, corresponding to the general admixture trend of East Asia. Over 70% of extant Korean genetic diversity is explained to be derived from such a recent population expansion and admixture from the South.

## Introduction

The 1000 Genome Project (1KGP) showed that East Asians displayed a common genetic bottleneck with non-African humans around the last glacial maximum (1000 Genomes Project Consortium et al. 2015). However, the 1KGP project includes only five EA populations failing to fully represent EA genome structures. In 2009, the HUGO Pan-Asian Consortium (PASNP) confirmed a general concordance between linguistic and genetic affiliations (HUGO Pan-Asian SNP Consortium et al. 2009). Most recently, the Asian diversity project showed a correlation between geographical coordinates and genetic structure in Asia (Liu et al. 2017). Although Koreans are similar to the Chinese, the PASNP, 1KGP, and Asian diversity projects cannot fully explain the detailed makeup and peopling of the Korean Peninsula.

Koreans belong to the Altaic language group and are known to be homogeneous in Northeast Asia along with the Chinese and the Japanese. There are ∼85 million Koreans in total (51 mils. South and 25 mils. North Koreans, and 7 mils. outside of the Korean Peninsula) unified by shared ethnic and linguistic traits. There are currently several hypotheses on the origins of the Korean. The Korean Y-chromosome haplogroup (O2b-SRY465) suggests the ancestors of the proto-Koreans are related to the people who inhabited northeastern China during the Neolithic (9,900–10,000 years BP) and Bronze (3,450–2,350 years BP) Ages (Kim et al. 2011). On the other hand, mitochondrial DNA (mtDNA) shows that Koreans display a very typical East Asian (Jin et al. 2009). Previous population studies have revealed that Koreans have not undergone any severe genetic bottlenecks and primarily consist of two genetic components (Takeuchi et al. 2017). One is strongly associated with China, but the other is less clear. Therefore, uncovering the exact genetic makeup of Koreans has not been carried out at a whole-genome scale using both present-day and ancient genomes.

Paleogenomics is a powerful tool to reveal the exact genetic lineages and affinities that cannot be resolved with present-day populations alone because frequent and complex genetic exchanges occur with or without cultural and linguistic exchanges. Archeological data unearthed in Korea provide the proto-Korean chronology and prehistories of the Korean Peninsula. The oldest archaic relics, such as the Acheulean axes, that have been found in South Korea date back hundreds of thousands of years, however, human bone preservation is poor due to the acidic soils and cannot acquire any ancient genetic data (Norton 2000). The earliest hominid evidences in the Peninsula date to be between 400,000 and 600,000 years ago (YA) (Park 1992). In spite of the claims about human bones in North Korea (Norton 2000; Bae and Bae 2012), these paleoanthropological materials are rare in Korea. Therefore, it is only possible to infer the exact Korean ethnic origins through ancient genomes found in the nearby regions, such as Devil’s Gate in Russian Far East (8,000 years BP) (Siska et al. 2017) and Tianyuan cave, Beijing (40,000 years old) (Yang et al. 2017). Fortunately, Neolithic to Iron Age ancient genomes from Southeast Asia (SEA) have become available recently (Lipson et al. 2018). Such ancient genomes, taken from a wide geographic and temporal distribution, should allow us to answer when and how the genomes of Southeast Asia contributed to the genetic makeup of Koreans.

## Materials and Methods

### Data Set

A total of 88 Korean samples were used that are available from the KoVariome database (Kim et al. 2018) (supplementary table S1, Supplementary Material online) and 208 worldwide present-day individual samples were collected: 13 African, 4 American, 26 European, 7 Oceanian, 5 Central Asian, 43 East Asian, 31 North Asian, 36 South Asian, 22 West Asian, and 21 Southeast Asian (supplementary table S2, Supplementary Material online). We collected and added six EA and nine SEA individuals (supplementary table S2, Supplementary Material online). We merged the whole-genome sequence (WGS) data with the human origin SNP panel data set (Lazaridis et al. 2014) including six Korean samples’ genotype information generated from this panel. A total of 155 ancient genomes were collected (supplementary table S3, Supplementary Material online). Our sample data were chosen to abundantly reflect our target Asian populations and resolve the genetic relationships between Koreans and other populations. All the 88 Korean samples were collected and sequenced according to the guidelines set by the Institutional Review Board (IRB) of the Genome Research Foundation (GRF) (supplementary table S1, Supplementary Material online). Informed consent for study participation was acquired from all participants by the Korean Life Ethics bill, and all experimental protocols were approved by the GRF IRB. We uploaded them on a web site Asian Genome Data for Korean Origin (http://variome.net/Asian_Genome_Data_for_Korean_Origin, last accessed April 17, 2020).

### Whole-Genome Sequencing and Genotyping

Samples were subjected to WGS and genotyping (supplementary table S2, Supplementary Material online). Genomic DNA was extracted using a QIAamp DNA Blood Mini Kit (Qiagen, CA) and 69 WGS libraries were constructed using TruSeq DNA sample preparation kits (Illumina, CA). Sequencing was performed using Illumina HiSeq sequencers following the manufacturer’s instruction. Low-quality reads were removed by NGSQC-toolkit (ver 2.3.3) with “-l 70 and –s 20” options (Patel and Jain 2012). Filtered reads were aligned to the human reference genome (hg19) using BWA-MEM (ver. 0.7.8) (Li and Durbin 2009). We further removed PCR duplicates using MarkDuplicates in Picard (ver. 1.9.2, http://broadinstitute.github.io/picard/, last accessed April 17, 2020) and conducted IndelRealigner and BaseRecalibration using GATK (ver. 2.3.9) (McKenna et al. 2010). We predicted individual single-nucleotide variants using GATK UnifiedGenotyper (McKenna et al. 2010) with “–heterozygosity 0.0010 -dcov 200 -stand_call_conf 30.0 -stand_emit_conf 30.0” options. To confirm artifacts in the variants merging from various resources which can occur during the production process caused by different sequencing platforms, alignment algorithms, and genotype callers, WGS-based variants were merged with the six Koreans’ genotypes generated from the human SNP panel data (Lazaridis et al. 2014). Finally, we pruned the panel with linkage disequilibrium information using plink with “–indep-pairwise 200 25 0.4” option (Purcell et al. 2007).

### Haplotype Analysis

Korean haplotypes were analyzed with YFitter (Jostins et al. 2014) for Y-chromosome and haplogrep (Kloss-Brandstatter et al. 2011) for mtDNA haplotypes (supplementary table S1, Supplementary Material online). To analyze the mtDNA haplotypes of the ancient genomes, we downloaded mitochondrial BAM files of ancient genomes via the European Nucleotide Archive with accession ID of PRJEB14817, PRJEB24939, and PRJEB9021 and GenBank with accession ID of KC417443.1 for the Tianyuan mitochondrion. Consensus sequences of ancient and modern mitochondrial genomes were generated by SAM tools with minimal depth 5. Then, multiple sequence alignment of the consensus sequences was performed by MUSCLE. The phylogenetic tree was constructed by MEGA7 with a Gamma distribution model and pairwise deletion for gap treatment. Divergence time between nodes was calibrated by MEGA7 with the four previously suggested calibration points for A (41,504–51,765), B (35,360–44,929), C (29,615–42,453), and D (41,610–52,388) (Bonatto and Salzano 1997).

### Genomic Clustering

We used CHROMOPAINTER to infer “chromosome chunks” for each individual for fineSTRUCTURE (Lawson et al. 2012) analysis and clustered 88 Koreans (supplementary table S1, Supplementary Material online) and 208 present-day individuals (supplementary table S2, Supplementary Material online) into 64 genetic groups (supplementary figure 1, Supplementary Material online). The fineSTRUCTURE produced a homogeneous group of 88 Korean individuals (supplementary figure 2, Supplementary Material online). In total, we reclustered 185 present-day genomes and 6 Korean genomes using CHROMOPAINTER and fineSTRUCTURE (Lawson et al. 2012). Using these individuals, we implemented ADMIXTURE (ver. 1.23) (Alexander et al. 2009) with *K *=* *2–14 (supplementary figure 3, Supplementary Material online). We generated a dendrogram with each of the ADMIXTURE result (K = 2–14) using the hcluster function in R. We evaluated the consistency of the ADMIXTURE and fineSTRUCTURE results by calculating correlation using the “cor.dendlist” function with the “cophenetic” method in the “dendextend” package in R (supplementary figure 4, Supplementary Material online). It showed the highest correlation when *K *=* *10 (corr. = 0.78). We used the admixture result of *K *=* *10, which best represents the genetic cluster analyzed by fineSTRUCTURE. We performed a principal component analysis (PCA) analysis conducted with EIGENSOFT (ver. 6.0.1) smartpca (Patterson et al. 2006).

### Admixture Time Estimation

We implemented the ALDER program (Loh et al. 2013) to estimate the admixture time of Korean using the Korean itself as one reference population. We used filtering criteria of a genotype rate >99%, MAF > 0.01, and Hardy–Weinberg equilibrium *P* value > 0.000001.

### The Genetic Affinity between the Ancient and Present-Day Populations

To investigate the genetic relationship between populations of interest, we used the *D* and outgroup *f3* statistic framework by using ADMIXTOOLS (Patterson et al. 2012). The genetic affinity between the ancient and present-day populations was measured with the outgroup *f3* statistic using the following notation: *f3*(*X*, *Y*; Yoruba), where *X* and *Y* are ancient and present-day populations, respectively. To better represent the genetic association of the present-day population against a focal ancient genome, we applied a scaled *f3* statistic by *f3*_scaled_ = (f3 − *m*)/(*M* − *m*), where *m* and *M* represent the minimum and maximum *f3* statistic (fig. 2*A* andsupplementary figure 5, Supplementary Material online). To cluster ancient genomes in this study, we analyzed a pairwise outgroup *f3* statistic with a form of *f3*(*X*, *Y*; Yoruba). In this analysis, both *X* and *Y* were ancient genomes.

### Admixture Model Construction

To construct an admixture model depicting the historical genetic makeup of Koreans and other Asians, we fitted the SNP panel to the admixture models with the qpgraph program (Patterson et al. 2012) based on results from *D*-statistics and *f*_3_ statistics in our study. We first set the skeleton for the admixture model as Tianyuan, Onge, and Ami by adapting a previous study (McColl et al. 2018) (worst-fitting *Z* = 0.044). Then, we added Kinh which has a high admixture F3 score with Devil’s Gate to Koreans (worst-fitting *Z*=−3.887) and then to Devil’s Gate, Ulchi, Koryak, Mixe, and MA1 (worst-fitting *Z* = 3.317). Finally, Koreans, Han, and Japanese have been added to model the suggested admixture of East Siberians (E_si_) and East Asians b (EA_b_) (worst-fitting *Z* value of −3.686). We manually calibrated the final model with a time point which was estimated using the ALDER results.

## Results and Discussion

### Korean Genetic Structure

**Fig. 1:**
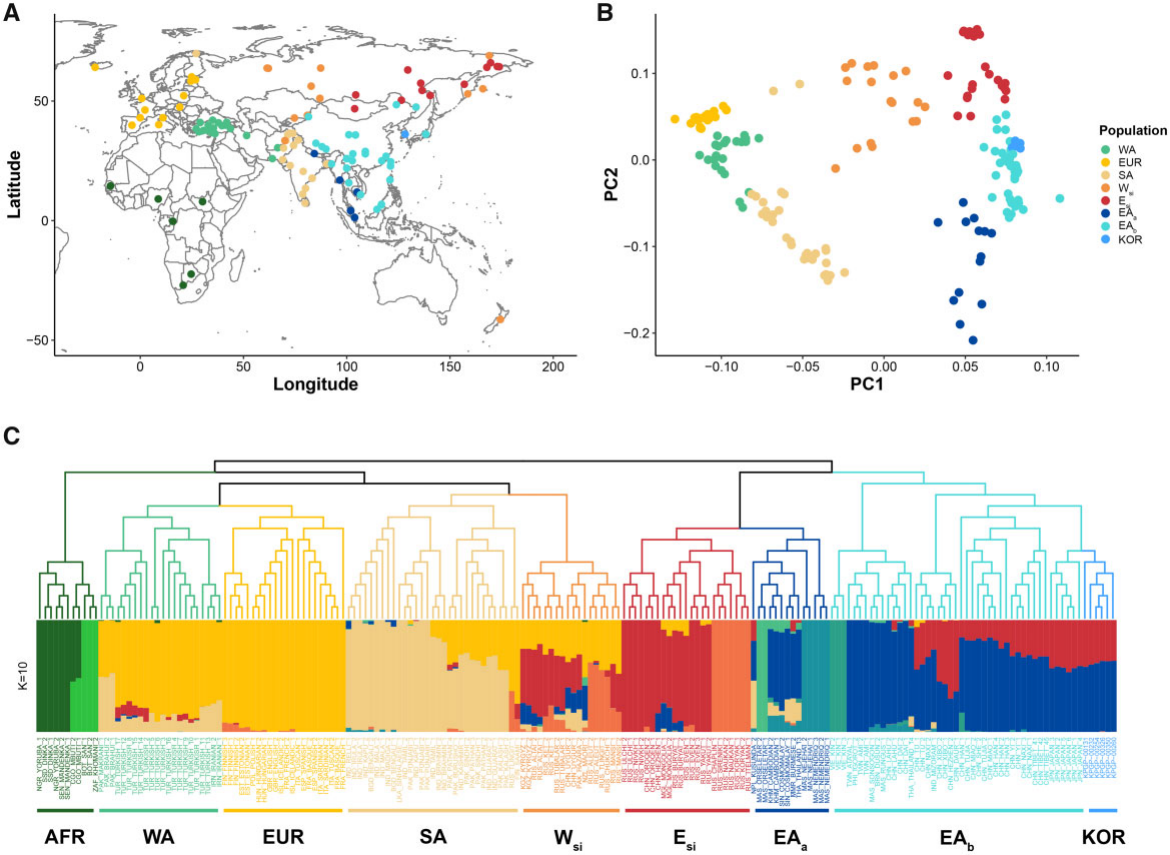
—Genetic clustering of the present-day populations. (*A*) Illustration of the geographical distribution of the 91 populations analyzed in this study. Each circle highlights a genetic cluster from (*B*). (*B*) Principal component analysis (PCA) of the 185 individuals using 199,629 linkage disequilibrium pruned SNPs in the 109 present-day populations. (*C*) Genetic clustering of present-day populations analyzed by fineSTRUCTURE (Lawson et al. 2012) (top) and ADMIXTURE (Alexander et al. 2009) (bottom). Names of the genetic clusters are given underneath the admixture group names.

To infer the genetic association between the 88 Koreans (supplementary table S1, Supplementary Material online) and our selected neighboring populations, we collected with WGS from 185 contemporary individuals belonging to 91 populations (fig. 1*A* and supplementary table S2, Supplementary Material online). We included people from 21 and 31 Southeast Asian and North Asian ethnic groups, respectively, from which Koreans could have originated. We predicted an average of 1.5 and 2.6 mega homo- and heterozygous single-nucleotide variants from each individual, respectively (supplementary table S2, Supplementary Material online). We merged WGS-based SNPs with the human origin SNP panel data set and finally produced 199,629 autosomal SNPs for genetic comparison. To infer the genetic structures of the Korean ethnic group, we clustered 94 Koreans, including 6 published Koreans genotyped with SNP chip, by applying the CHROMOPAINT and fineSTRUCTURE (Lawson et al. 2012) programs. These algorithms clustered 279 individuals into 64 homogeneous groups according to the haplotype patterns shared by the individuals (supplementary figure 1, Supplementary Material online). This analysis showed eight global haplotype patterns: Africans (AFR), West Asians (WA), Europeans (EUR), South Asians (SA), West Siberians (W_si_), East Siberians (E_si_), and two groups of East Asians (EA_a_ and EA_b_) (supplementary figure 2, Supplementary Material online), which reflect both geographic and genetic relationships (fig. 1*A*). The group of EA_b_ consists mainly of Korean, Chinese, Japanese as well as Austroasiatic speakers in Southeast Asia and EA_a_ contains several ethnic minorities of Southeast Asia. We first confirmed a genetically homogeneous ethnic group of Koreans by showing a single clade in the fineSTRUCTURE tree (supplementary figure 2, Supplementary Material online). This homogeneity is also consistent across chip-based and WGS-based data, suggesting that there is no technical bias in the sequencing platform or the SNP prediction algorithm. In the PCA, both the Koreans and EA_b_ fell between the EA_a_ and E_si_ populations (fig. 1*B*), consistent with other previous studies (Kim and Jin 2013; Wang et al. 2018). We reanalyzed fineSTRUCTURE and ADMIXTURE (Alexander et al. 2009) with 6 randomly sampled Koreans and 185 global populations, to compare Korean’s genetic components without sampling bias (fig. 1*C*). Consistent with the PCA result, the fineSTRUCTURE tree showed Koreans formed a homogeneous clade with most of the EA populations represented by EA_b_ and their sister groups were composed of E_si_ and EA_a_ (fig. 1*C* top). We also analyzed genetic ancestry assuming ancestral groups from *K *=* *2 to *K *=* *14 in the ADMIXTURE analysis (Alexander et al. 2009) (supplementary figure 3, Supplementary Material online). From *K *=* *5, it showed two genetic components, red and blue, were admixed in Koreans which were dominated in the E_si_ and EA_a/b_ populations, respectively; although, these ratios were slightly different depending on the number of ancestral groups (*K*). The dendrogram correlation analysis showed the greatest consensus between the fineSTRUCTURE clades and ADMIXTURE results at *K *=* *10 (supplementary figure 4, Supplementary Material online). At *K *=* *10, we observed 38% and 62% of the E_si_ and EA_a/b_ genetic components in the Koreans, respectively (fig. 1*C*). Comparing admixture rates among the EA_b_ populations, both the Korean and Japanese populations showed very similar levels of genetic admixture rates, consistent with their sister groups in the fineSTRUCTURE tree (fig. 1*C*). Takeuchi et al. (2017) reported a high degree of genetic similarity between the Korean and mainland Japanese and the estimated admixture date of the EA-wide genetic component to Japan was in the Yayoi period (3,000–1,700 years BP). The Chinese also have similar genetic compositions to the Korean and Japanese; however, their admixture rates differed depending on geographic region. Overall, we conclude that genetic admixture events occurred first between the Southeast Asians and Chinese outside Korea and Japan and then spread, rather than occurring separately in Korea or Japan locally. It is also possible that such a recent genetic admixture was a broad phenomenon, happening concurrently all across EA driven by a population expansion caused by the agricultural, economic, and technological advances of the last 4,000 years (Lipson et al. 2018).

### The Gene Flow Neolithic Age Devil’s Gate Ancestry to Korean People

**Fig. 2:**
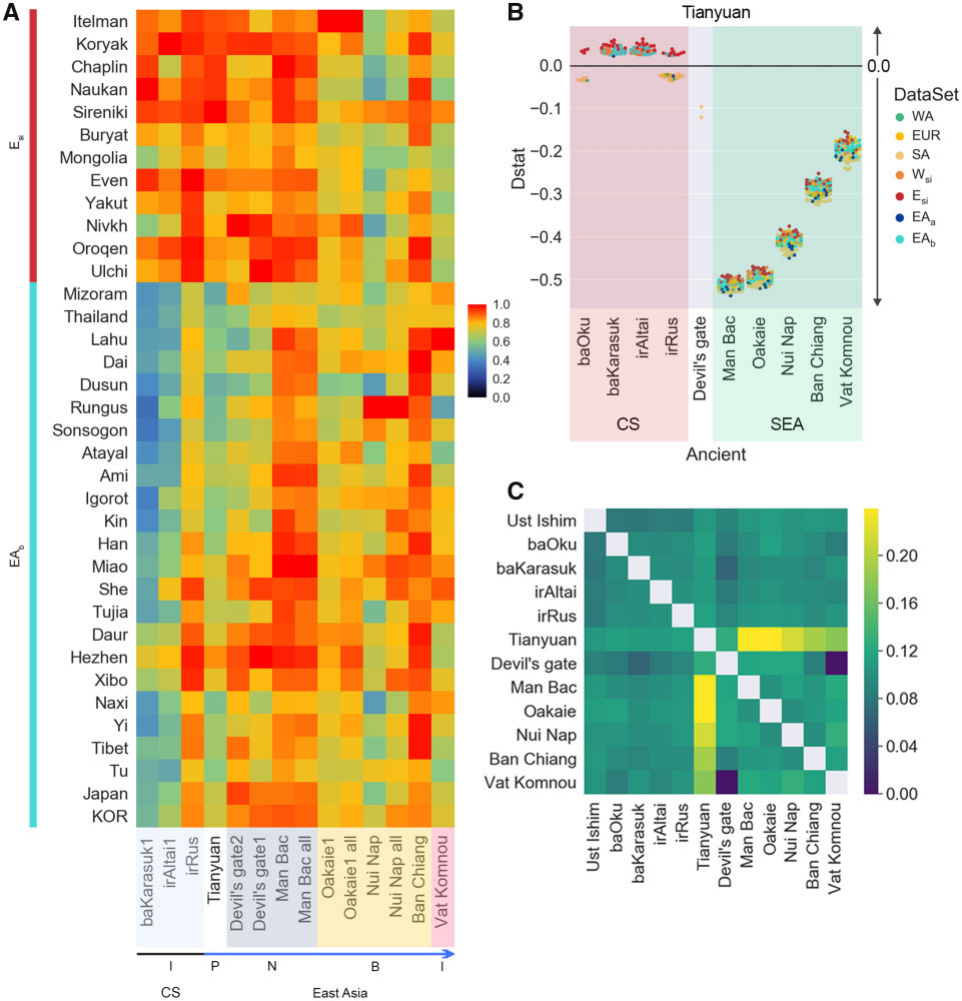
—Genetic association between the ancient and present-day populations. (*A*) Outgroup *f3* statistics with the form of *f3*(*X*, *Y*; Yoruba), where *X* and *Y* are ancient and present-day populations, respectively. We scaled *f3* statistics between 0 and 1. In the heat map, black indicates that the *f3*_scaled_ value is close to 0 and red indicates the value is close to 1. For ancient genome *X* (on rows), the scaled *f3* statistic for a given cell is calculated by *f3*_scaled_ = (f3−*m*)/(*M*−*m*), where *m* and *M* represent the minimum and maximum *f3* statistic. Therefore, the smallest *f3* in each column has *f3*_scaled_-statistic = 0 (black) and the largest has *f3*_scaled_-statistic = 1 (red). We ordered ancient genomes in the *x* axis according to the time scale. We also separated Central Steppe (CS) ancestry (black arrow) (de Barros Damgaard et al. 2018) and Chinese and Southeast Asian ancestry genomes (blue arrow) (Lipson et al. 2018). P on the bottom bar, Pleistocene hunter-gatherers; N, B, and I, Neolithic hunter-gatherer, Bronze, and Iron age, respectively. Overall, data for these statistics are found in supplementary figure S5 and table S4, Supplementary Material online. (*B*) *D*(Yoruba, Tianyuan; *X*, *Y*), where *X* and *Y* are ancient and present-day populations, respectively. We represented only the absolute |*Z*-score| >3. The spot colors represent the individual’s genetic cluster in figure 1*C*. The *x* axis represents ancient genomes that have a genetic affinity with East Asia (EA) and East Siberia (E_si_) populations, shown in figure 1*C*. The overall data on 115 ancient genomes for this *D*-statistic are found in supplementary figure S6, Supplementary Material online. (*C*) Outgroup *f3* statistics among ancient genomes with the form of *f3*(*X*, *Y*; Yoruba). Both *X* and *Y* were ancient genomes. The overall ancient clustering is represented in supplementary figure S7, Supplementary Material online.

To reveal past genetic exchanges contributing to the current Koreans and their neighboring populations, we collected 115 ancient genomes from across the world (supplementary table S3, Supplementary Material online), consisting of 4 Pleistocene hunter-gatherers, 13 Holocene hunter-gatherers, 20 Early Neolithic, 10 Mid Neolithic, 10 Late Copper Age, 9 Late Neolithic, 20 Early Bronze Age, 4 Mid Bronze Age, 2 Late Bronze Age, and 12 Iron Age ancient genomes distributed across European and Russian regions (supplementary table S3, Supplementary Material online). The time scale of these ancient genomes was categorized by referring to previous research (Haak et al. 2015). In addition, we included the Tianyuan genome from northern China (Yang et al. 2017), two ancient genomes unearthed from the Devil’s Gate cave near North Korea (Siska et al. 2017), and eight ancient genomes from Southeast Asia dating from the Neolithic to the Iron Age (Lipson et al. 2018), making a total of 115 genomes. We measured levels of pairwise genetic affinity among the ancient and present-day genomes by using outgroup *f3*-statistics, with a form of *f3*(ancient, present-day; Yoruba) (Patterson et al. 2012). This analysis calculates the global landscape of the genetic associations between ancient and present-day genomes (supplementary figure 5 and table S4, Supplementary Material online). The *f3*_scaled_-statistics showed that the ancient Tianyuan individual (40,000 years BP from China) shares more alleles with present-day Siberians (E_si_ and W_si_) and East Asian (EA_b_) populations than with other present-day populations such as European, West-, and South Asians (supplementary figure 5, Supplementary Material online). It suggests Tianyuan is the basal genetic component of the East Eurasian and East Asian lineage. We also observed that present-day E_si_ and EA_b_ populations had significant genetic affinities with ancient Southeast Asians (ancSEA), Devil’s Gate, and Bronze and Iron age ancients who lived in central steppe regions (ancCS) (fig. 2*A* and supplementary table S4 and figure 5, Supplementary Material online). Based on these genetic affinities, we deduced the genetic founders of the Koreans by comparing the Tianyuan-derived alleles shared with these ancients and present-day populations. We applied *D*-statistics in the form of *D*(Yoruba, Tianyuan; *X*, *Y*), where *X* and *Y* were ancient and present-day populations, respectively (fig. 2*B* and supplementary figure 6, Supplementary Material online). Tianyuan shares more derived alleles with ancSEAs than with any present-day populations (fig. 2*B*), suggesting ancSEAs directly come from the Tianyuan lineage. Neolithic Devil’s gate and present-day population (E_si_ and EA_a/b_) showed a similar amount of Tianyuan’s genetic ancestry by showing *D*(Yoruba, Tianyuan; Devil’s Gate, E_si_ or EA_a/b_) ≈ 0. It suggests Neolithic Devil’s gate (Northern part of Korea) is possible to be admixed with another genetic component. In addition, Tianyuan’s genetic ancestry had a significantly higher level of genetic affinity with W_si_, E_si_, and EA_b_ populations than with ancCS (fig. 2*B*). It suggests ancCS were possibly generated from other genetic compounds. The genetic clustering of ancient genomes also confirmed the highest genetic affinity of Tianyuan in Man Bac and a slight reduction of this affinity in other ancSEAs over time (fig. 2*C* and supplementary figure 7, Supplementary Material online). This evidence suggests ancSEA received an additional genetic component over time, consistent with Man Bac having the highest affinity toward Tianyuan.

We examined Tianyuan’s genetic affinities for E_si_ and EA_a/b_ using *D*-statistic in the form of *D*(Yoruba, Tianyuan; E_si_, EA_a/b_) (supplementary figure 8, Supplementary Material online). In these statistics, the Tianyuan genome showed a higher level of genetic affinity with present-day E_si_ than Southeast Asians. However, several EA_b_ (Korean, Japanese, and south Chinese) populations showed similar levels of affinity with Tianyuan-derived alleles to the E_si_ populations and were equally distant to Tianyuan lineage. This suggests Devil’s Gate ancients and present-day E_si_ and several EA_b_ populations were subject to similar genetic influences over time and are expected to be a single clade since they are all separated originally from the Tianyuan lineage. These lines of analysis reveal that the basal ancient of the Tianyuan genome was separated in the Neolithic or pre-Neolithic era and independently affected current Koreans.

### The Ancient Gene Flow Making Up the Korean Ethnic Group

**Fig. 3:**
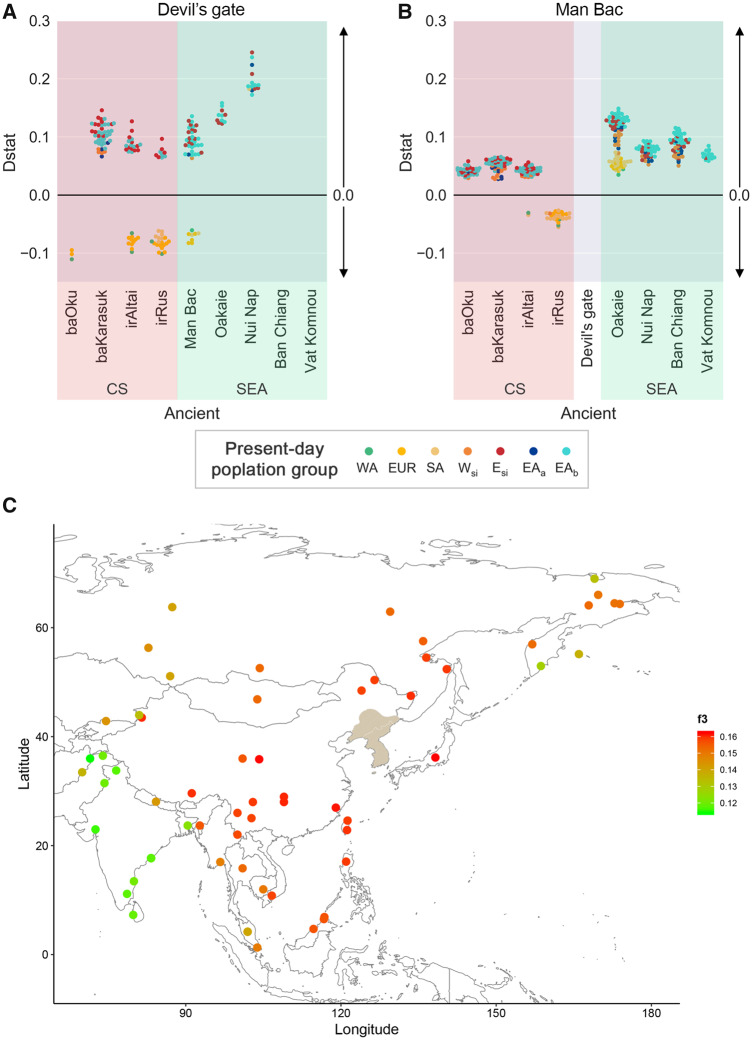
—Bronze and Iron Age gene flows making up the Korean. Ancestry analysis from Neolithic ancients to present-day populations with forms of (*A*) *D*(Yoruba, Devil’s gate, ancient, present-day population), (*B*) *D*(Yoruba, Man Bac, ancient, present-day population). We represented only the |*Z*-score| >3 for each *D*-statistics. The positive values represent genetic ancestry to present-day populations and the negative values represent genetic ancestry to ancients at the bottom. Raw data for these analyses are represented in supplementary tables S5 and S7, Supplementary Material online. The CS represents ancient genomes generated from central steppe regions (de Barros Damgaard et al. 2018). (*C*) Koreans’ genetic affinity with neighboring ethnic groups with outgroup *f3* statistics, a form of *f3*(Korean, *Y*; Yoruba). The spot colors represent the genetic affinity of *f3*-statistics. The overall ancient clustering is represented in supplementary table S8, Supplementary Material online. The predicted historical Korean territories are given in ocher which referenced the website of “About Korea” (http://www.korea.net/AboutKorea/History/Three-Kingdoms-other-States, last accessed April 17, 2020).

We focused on the gene flow from the Neolithic ancients into the Korean and EA populations. Based on the Tianyuan’s gene flow into Neolithic ancients and present-day populations, we hypothesized that either the Neolithic ancient genome contributed to the genetic ancestry of Korean or EA populations independently, or a second gene flow could have occurred (fig. 2*B*). First, we investigated gene flow from two Neolithic ancients to Koreans and EA populations, with a form *D*(Yoruba, Devil’s Gate/Man Bac, ancient, present-day population). It showed Devil’s Gate genomes shared more derived alleles with most of the present-day E_si_ and EA_b_ populations than with Neolithic Man Bac in Vietnam (fig. 3*A* and supplementary table S5, Supplementary Material online). From the Devil’s Gate genome near North Korea, we observed these present-day populations are equivalent to the genetic relationship with Ban Chiang and Vat Komnou ancients who are ancestors of Austroasiatic speakers (Lipson et al. 2018). In addition, we observed local genetic transitions from Oakaie (Late Neolithic and Bronze Age in Myanmar) and Nui Nap (Bronze Age in Vietnam) to EA populations (supplementary table S5, Supplementary Material online). Several E_si_ and EA_b_ populations, such as Korean, Japanese and several Chinese (Hezen, and She), and Russian (Ulchi) ethnic group, still had dominant genetic contributions from Devil’s Gate compared with Oakaie and Nui Nap ancients. This suggests that local genetic differences observed in present-day EA_a/b_ populations (fig. 1*C*) were influenced by a new genetic influx from the Bronze Age to Iron Age in Southeast Asia. We also observed *D*(Yoruba, Devil’s gate, baOku, present-day E_si_ or EA_b_) ∼0 (fig. 3*A*) and *D*(Yoruba, baOku, E_si_, EA_b_) ∼0 (supplementary table S6, Supplementary Material online). According to these statistics, the baOku genomes are equally closely related to present-day E_si_ and EA_b_ populations, which is different from the dominant ancestry of the E_si_ populations in baKarasuk (Iron Age in Russia) and irAltai (Iron Age in Russia). Unlike the Devil’s Gate’s ancestry, the Neolithic Man Bac shares more derived alleles with most of the present-day E_si_ and EA_b_ populations than either the Bronze Age ancSEAs (Oakaie, Nui Nap, Ban Chiang) or ancCSs (baOku, baKarasuk, irAltai) (fig. 3*B* and supplementary table S7, Supplementary Material online). This suggests the Neolithic Man Bac is the basal ancestry for the present-day E_si_ and EA_b_ populations. No genetic drift was observed from Neolithic Man Bac to Devil’s Gate ancient and present-day populations (fig. 3*B*). We also analyzed genetic associations of ancCS to other ancients and present-day populations with a form of *D*(Yoruba, ancCS; ancient, present-day populations) (supplementary figure 9, Supplementary Material online). It inferred that present-day E_si_ and EA populations and ancSEA are equally related to ancCS by sharing similar levels of ancCS-derived alleles. It is an agreement with genetic admixture patterns of Asian ancestry in CS ancients (Allentoft et al. 2015; Damgaard et al. 2018). It supports genetic admixture between ancCS and present-day EA populations, however, it cannot explain how and how many events the ancCS influence toward EA occurred. We also observed the first evidence of the genetic divergence of Vat Komnou and several EA_b_ (Southeast Asian and Southern China) populations from Man Bac (fig. 3*B* and supplementary table S7, Supplementary Material online). This supports the idea that these ancients are new genetic resources that genetically influenced EA (fig. 2*A*). We observed several possible ancient founders by *D*-statistics, however, it could not clearly resolve the current genetic makeup of Korean. To resolve the genetic relationship of the genetic makeup of Korean, we additionally analyzed the admixture pattern of the ancient/present-day Southeast Asians and Devil’s Gate ancients to Koreans with admixture *f3* statistics (table 1). Notably, the combinations of the Devil’s Gate genome and ancSEAs better represent the current Koreans than those of Devil’s Gate and modern Southeast Asians. Specifically, we observed the lowest admixture *f3*-statistics when source 1 was Vat Komnou (Iron Age in Cambodia), followed by Nui Nap (Bronze Age in Vietnam). In a previous study, Nui Nap was a new genetic component close to present-day Vietnamese and Dai but not the ancestors of Austroasiatic speakers (Lipson et al. 2018). Meanwhile, next ancSEAs with lowest admixture *f3*-statistics were Ban Chiang and Man Bac who are also ancients of Austroasiatic speakers. In order to investigate whether the ancSEA genetic components migrated into Korea, we analyzed the Koreans’ genetic affinity with present-day populations by outgroup *f3*-statistics with a form of *f3*(Korean, present-day populations; Yoruba) (fig. 3*C* and supplementary table S8, Supplementary Material online). It showed the group with the highest genetic affinity with the Koreans were the Japanese. The southern Chinese (Han, and She) had a higher genetic affinity with Koreans than the present-day Lau or Vietnamese, which is consistent with the admixture results (fig. 1*C*). This suggests that the genetic components of South Chinese were transferred into Korea after admixing with Vat Komnou and Nui Nap ancestries (fig. 3*C*). These lines of evidence support the conclusion that populations who carried Devil’s Gate and Man Bac genomes admixed throughout the EA_b_ and E_si_ regions until the Neolithic period, probably accompanied by the climate changes and barriers. After the Bronze Age, the admixed genetic ancestry of the Vat Komnou and Nui Nap migrated to Korea due to rapid cultural and technological advances.

**Table 1:** **Admixture *f3* Statistics** ^a^

Source1	Source2	Avg. *f3*	Min. *f3*	Max. *f3*
Vat Komnou	Devil’s gate2	−0.192366	−0.22219	−0.173976
Nui_Nap	Devil’s gate1	−0.13199	−0.13199	−0.13199
Ban_Chiang_all	Devil’s gate1	−0.127784	−0.127784	−0.127784
Ban_Chiang	Devil’s gate2	−0.118145	−0.118145	−0.118145
Nui_Nap_all	Devil’s gate1	−0.10339	−0.10339	−0.10339
Man_Bac	Devil’s gate2	−0.055678	−0.056621	−0.054339
Atayal_EA	Devil’s gate2	−0.038359	−0.04107	−0.035966
Ami_EA	Devil’s gate2	−0.0380293	−0.040296	−0.036663
Lahu_EA	Devil’s gate2	−0.036503	−0.039709	−0.034341
Kinh_EA	Devil’s gate2	−0.034616	−0.036383	−0.031549
Thai_EA	Devil’s gate2	−0.0334685	−0.035207	−0.03173
Dai_EA	Devil’s gate2	−0.032952	−0.033388	−0.032296
Cambodian_EA	Devil’s gate2	−0.032376	−0.032407	−0.032345
Tujia_EA	Devil’s gate2	−0.0314865	−0.032745	−0.030228
Han_EA	Devil’s gate2	−0.030894	−0.031301	−0.030493
She_EA	Devil’s gate2	−0.0303735	−0.031006	−0.029741
Miao_EA	Devil’s gate2	−0.03032	−0.03032	−0.03032
Yi_EA	Devil’s gate2	−0.030312	−0.030312	−0.030312

^a^ The notation of admixture *f3* statistic: *f3*(Source1, Source2; KOR) and only represented with |*Z*-score|>3.

### Korean Haplotype Analysis Reveals Multiwaves of Genetic Components

**Fig. 4:**
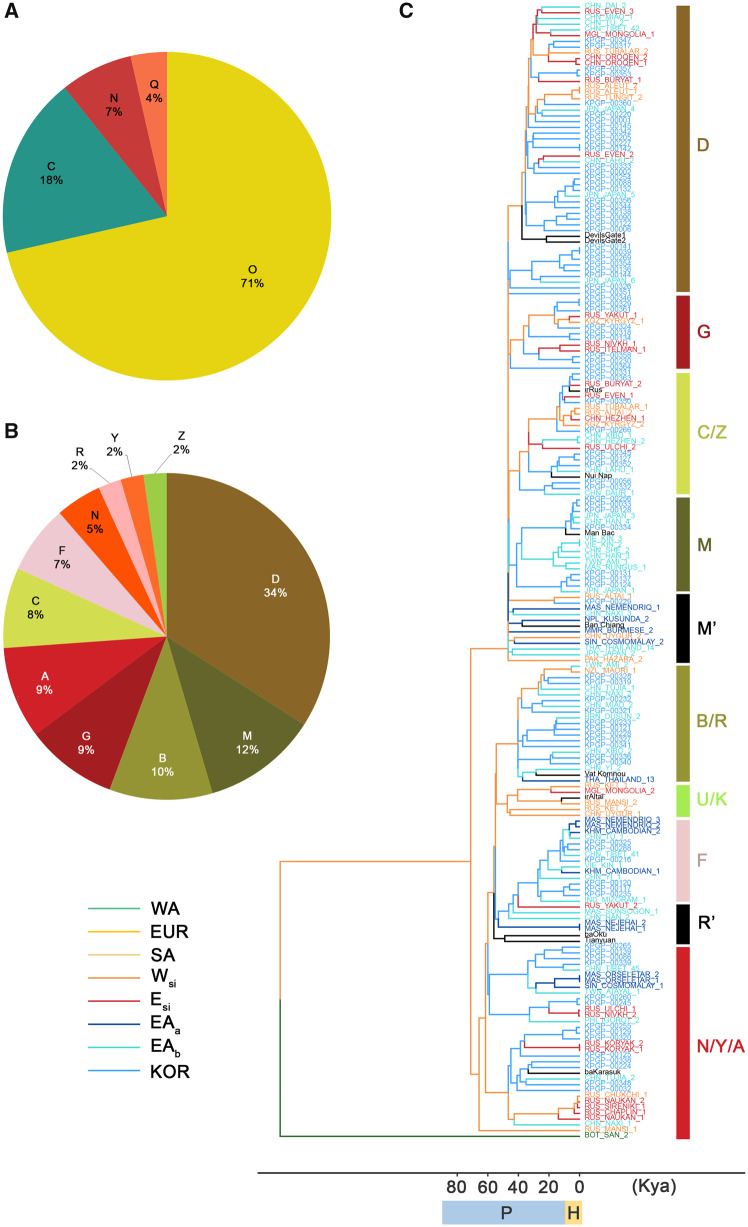
—Haplotype distribution in the Korean population. (*A*) Y-chromosome haplotypes from 55 male Koreans, (*B*) mtDNA haplotypes in 88 Koreans, and (*C*) a phylogenetic tree of mtDNA haplotypes constructed using the neighbor-joining method with bootstrap=1,000. We give the dominant mtDNA haplogroup clusters on the right of the tree. The ancient haplogroup is represented by M′ and R′. P, Pleistocene; H, Holocene.

We analyzed haplotype distributions using WGS data of 88 unrelated Koreans generated from the KoVariome database (Kim et al. 2018) (supplementary table S1, Supplementary Material online). Nonrecombining Y-chromosome analysis showed a significant proportion of the “O” haplogroup in 55 male Koreans, 29% “O2b” and 42% “O3” (fig. 4*A*). The next most frequent Y-chromosome haplogroup was “C” (18%). The Y-chromosome haplogroup distribution agreed with well-established Y-chromosome haplogroup “O” expansion and colonization within the Korean Peninsula (Kim et al. 2011). A comparison with the global Y-chromosome haplogroup distribution suggested that haplotype “C” is widespread in Siberia, whereas “O” haplogroups show a spatial distribution in Southeast Asia (Chiaroni et al. 2009; Karmin et al. 2015). This strongly suggests a dual origin for Korean males. In contrast to the Y-chromosome distribution, mtDNA haplotypes reflect a more complex genetic history (fig. 4*B*). The most frequent mtDNA haplotype was “D” (34%) and ten additional mtDNA haplogroups (“M,” “B,” “N,” “G,” “F,” “R,” “A,” “C,” “Y,” and “Z”) were identified with frequencies ranging from 23% to 2%. We constructed an mtDNA tree combining 11 ancients, and 99 present-day EA_a/b_ and Siberian (E_si_ and W_si_) mtDNAs (fig. 4*C*). We included 11 ancients in this tree who had relatively high-sequencing depth (supplementary table S9, Supplementary Material online). Similar to the global human-mtDNA phylogeny, our mtDNA tree shows two major clades, M′ and R′, dominantly distributed in EA populations (Soares et al. 2009). It also shows two mtDNA dispersions ∼40 and 20 ka, which account for 62% and 38% of the present-day Koreans, respectively. The earlier dispersed mtDNAs included “N/Y/A,” “D,” and “B/R” which were distributed to 16%, 34%, and 12% of Koreans, respectively. The mtDNA haplotypes of the “N/Y/A” and “D” were clades coclustered with present-day Siberians as well as the Devil’s Gate ancients, representing Eurasian ancestry. The “A” haplogroup was also frequently observed in the early and middle Bronze Age Okunevo peoples (Lipson et al. 2018), who were culturally associated with baKarasuk (Lipson et al. 2018). We also identified ancient mtDNA “R′” divergent into “B/R,” accounting for 12% of Koreans, that also expanded ∼40 ka. The root of this clade was Tianyuan, and also coclustered with Vat Komnou ancients and present-day Chinese, representing EA ancestry. This could explain the genetic influence of the Tianyuan on Korean genomes via ancSEA. These old mtDNA waves accounted for human migration in the late Pleistocene when the Yellow sea of Korea was land, therefore, the west coast of Korea was connected to the mainland of China. The later dispersed mtDNA haplogroups consisted of “G/C/Z,” “M,” and “F” which account for 19%, 12%, and 7% of Koreans, respectively. The “G/C/Z” clades coclustered with Siberians and Bronze Age Nui Nap in Vietnam. However, the genetic origin of the Nui Nap is still unknown. On the other hand, the mtDNA haplogroup “C” is frequently observed from the early and middle Bronze Age Okunevo peoples who lived in central steppe regions (Lipson et al. 2018). The mtDNA topology and haplotype frequency in Okunevo imply a genetic association between Nui Nap and central steppe ancients. Both of the “M” and “F” clades showed subsequent diversification from ancient mtDNA haplogroups of ancM (M′) ∼20 ka and ancR (R′) divergent in 60 ka, respectively. These clades explain southern waves of human migration by coclustering with EA_b_ populations. In particular, two ancients of Austroasiatic speakers, Man Bac and Ban Chiang, coclustered in the mtDNA “M” lineage (fig. 3*C*). It suggests that a subsequent expansion of this clade can be associated with the expansion of the Austroasiatic speaking population (Lipson et al. 2018). Haplotype analysis and the phylogenetic tree of the mtDNA support a continuous genetic influence from the north and south into Korea.

### Admixture Time Estimation for Koreans

**Fig. 5:**
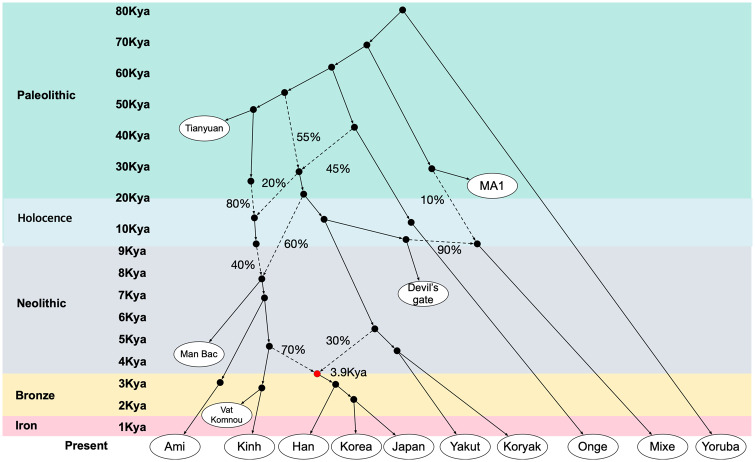
—Admixture tree model depicting the historical genetic makeup of Korean. A qpgraph (Patterson et al. 2012) fitted on an admixture model depicting the historical genetic makeup of Koreans and other Asians. We fitted the admixture tree model with ancient genomes associated with EA_b_ populations to make a model that could best explain the gene flow that makes up Koreans and hence the admixture model information for E_si_ ancestry has been simplified. Based on the *D*- and *f3* statistics and previous reports (Lipson et al. 2018), we set the skeletal tree (supplementary figure 10*A*, Supplementary Material online) and extended the model by adding ancient and present-day individuals (supplementary figure 10, Supplementary Material online). The average admixture time of Koreans is noted next to the red circle which was estimated by ALDER (table 2). Black circles represent ghost genomes in ancestral genetic lineages lacking any evidence for a time calibration and new groups may be added when more ancient populations are found and sequenced. Black lines represent the gene flow and dotted lines represent admixture events with the marked proportions estimated by qpgraph analysis.

We estimated the admixture time of Koreans using 286,222 SNPs and obtained significant prediction results from only three populations as references; Yakut, Han, and Japanese (table 2). The estimated admixture time was 5,482, 3,583, and 2,827 YA when we used the Koreans itself as one reference and Yakut, Han, and Japanese as the other comparison reference population, respectively. Our estimated admixture time with Japanese (97 generations away from the Japanese) is slightly earlier than the admixture date of the mainland Japanese (52 generations) estimated by Takeuchi et al. (2017). We summarized our model of the genetic influence by pre-Neolithic Tianyuan to Iron Age Vat Komnou on Koreans in figure 5. This model supported the above gene flows well, suggesting Koreans contain prehistoric genetic components derived from Devil’s Gate and Man Bac groups both of whom are divergent from Tianyuan ancestry. The Neolithic Man Bac genome dominantly inherited the genetic components of Tianyuan and showed its genetic components widely distributed in EA. However, the Bronze and Iron Age ancients, such as Oakaie, Nui Nap, and Vat Komnou, seem to have much altered genetic components of EA_b_ genomes (70%). This is consistent with the EA_b_ ancestry frequency in contemporary Koreans. This model generally describes well the gene flow among the three Northeast Asians; Korean, Chinese, and Japanese.

**Table 2:** Estimation of Admixture Date of Koreans

Population Group	Reference Population	No. of Sample	Admixture Time^a^		*Z*-Score	*P* Value
			Generation	Years		
E_si_	Yakut	20	189.05 (65.86–312.24)	5,482 (1910–9055)	3.01	1.3×10^−3^
EA_b_	Han	33	123.56 (72.05–175.07)	3,583 (2089–5077)	3.85	5.9×10^−5^
EA_b_	Japanese	29	97.47 (34.60–160.35)	2,827 (1003–4650)	3.71	1.0×10^−4^

^a^ The admixture time is shown in generations before the present. The number in the parentheses indicates 95% confidence interval of the generation and years.

## Conclusion

We analyzed the haplotype distributions of 88 Koreans compared with ancient and modern whole genomes and suggested two major haplotype expansion events. A comprehensive genome comparison confirmed that Koreans possess dual ancestral genetic components originating broadly from East Siberia (E_si_) and East Asia (EA_b_). Ancient genome comparisons revealed that the genetic makeup of Koreans can be best described as an admixture of the Neolithic Devil’s Gate genome in Russia and the Iron Age Vat Komnou in Southeast Asia. Our analyses of ancient and present-day populations suggest a long and gradual admixture model of two Neolithic founders, the Devil’s Gate founder in Russia and the founder from Tianyuan Cave in China. These two major components were admixing throughout East Siberia and East Asia for an extended time up until the Neolithic period. Subpopulations of current East Asians, as well as modern Koreans, were probably established by a later regional genetic transition during the Bronze Age. The peopling of Korea is most likely a part of large population expansion and the subsequent admixture events which occurred in East Asia, rather than a unique isolated event or migration. We think that this kind of recent rapid expansion and admixture could be general models for other East Asian and Southeast Asian populations in which Bronze and Iron Age populations expanded and admixed with other peripheral region populations. 

## Supplementary Material

evaa062_Supplementary_Data

## Notes

**Data deposition:** This project has been deposited at GenBank under the accession provided in supplementary table S1, Supplementary Material online.
