# Supplementary material for: The Origin and Composition of Korean Ethnicity Analyzed by Ancient and Present-Day Genome Sequences
Source: Genome Biol Evol. 2020 Mar 27;12(5):553–65. doi: 10.1093/gbe/evaa062 (PMC7250502; doi:10.1093/gbe/evaa062)
Supplement: evaa062_Supplementary_Data [file evaa062_supplementary_data.zip › Korean_Origin_FigureS_GBE_rev_20200313.docx]

**Supporting information**

**Supplementary Table S1. Haplotype distribution in 88 unrelated Koreans.**

**Supplementary Table S2. Whole-genome variome data of the 91 present-day populations analyzed in this study**

**Supplementary Table S3. Ancient genomes applied in this study**

**Supplementary Table S4. Global genetic affinity analyzed by outgroup *f3_scaled_*(ancient, present-day; Yoruba)**

**Supplementary Table S5. *D*-statistics with a form of *D*(Yoruba, Devil’s gate, ancients, present-day population)**

**Supplementary Table S6. *D*-statistics analyzing ancient gene flow to present-day population ^a^**

^a^ *D* statistics with a form of *D*(X, Y, Ancient, Yoruba) analysis, where X and Y were present-day E_si_ and E_ab_ individual having a high genetic affinity with Korean (Figure 2C)

**Supplementary Table S7. *D*-statistics with a form of *D*(Yoruba, Man Bac, ancients, present-day population)**

**Supplementary Table S8. Outgroup *f3*(Korean, present-day population, Yoruba) analysis**

**Supplementary Table S9. mtDNA depth for ancient genomes**

**
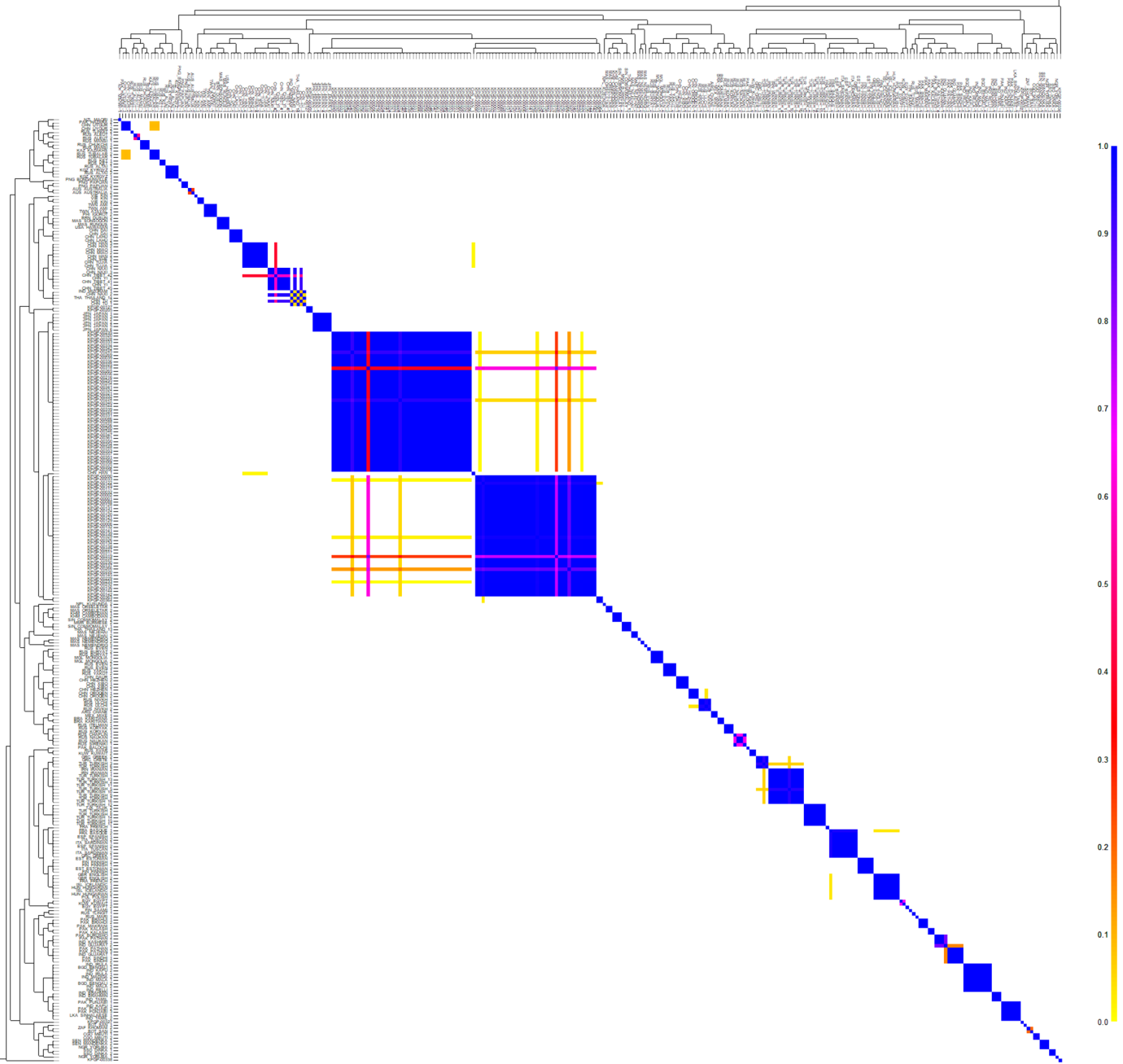
Supplementary Figure 1. FineSTRUCTURE analysis of 88 Koreans and 208 contemporary global individuals.**

**
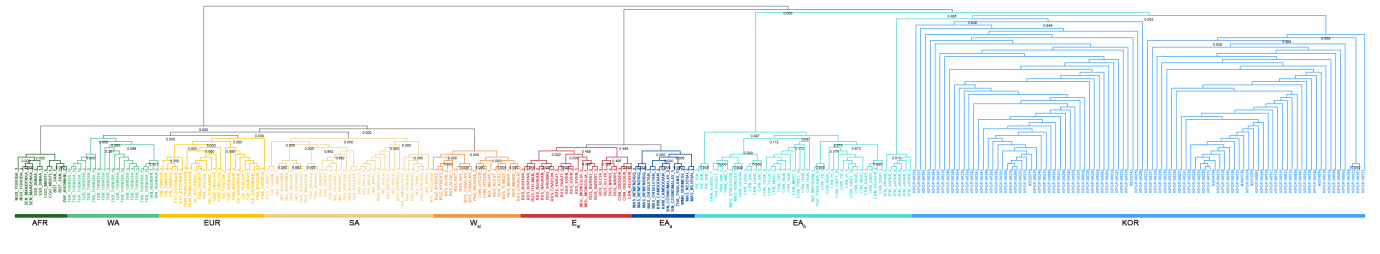
**

**Supplementary Figure 2. Global inference of the genetic structures.** The phylogenetic tree produced by the fineSTRUCTURE algorithm, which rotates clades according to the geographic associations without topological (branching structure) changes. The genetic distance from the assumed common ancestor is not mathematically scaled and it just shows the cluster topology.


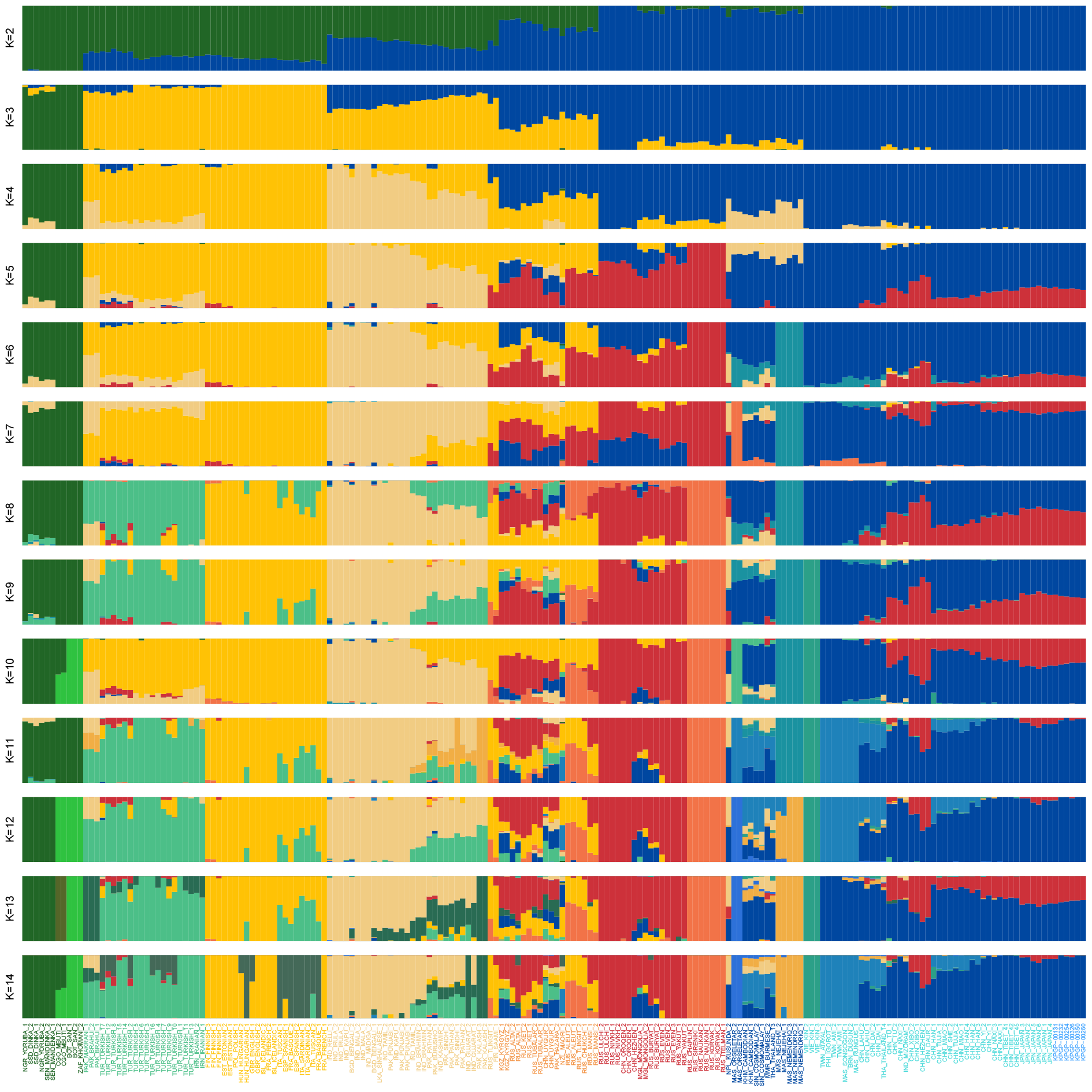


**Supplementary Figure 3. Global inference of the genetic structures after filtration (*K*=2-14)**


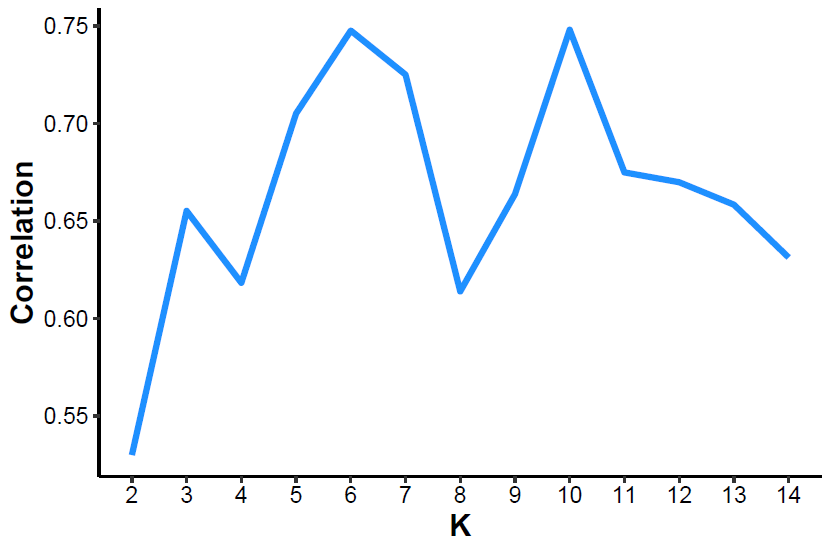


**Supplementary Figure 4. Dendrogram correlation between the fineSTRUCTURE clade and ADMIXTRUE (K=2-14) results.**

**
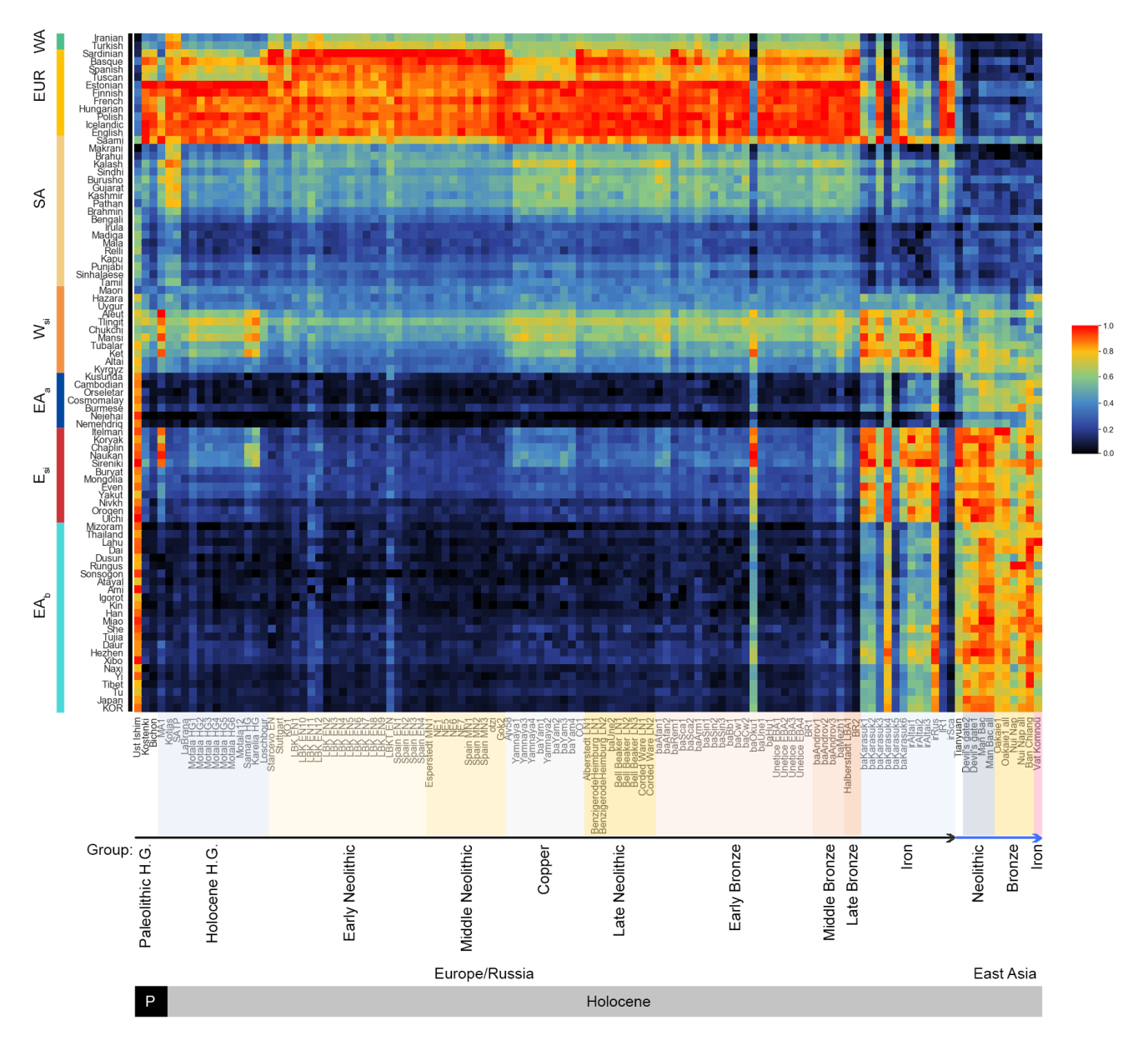
**

**Supplementary Figure 5. A global outgroup *f3* statistics between the ancient and present-day populations**

Outgroup *f3* analyses with the form of *f3*(X, Y; Yoruba), where X and Y are ancient and present-day populations, respectively. We scaled the *f3* statistics between 0 (black) and 1 (red) in the heat map. For ancient genome X (on rows), the scaled *f3* statistic for a given cell in that column is calculated by *f3_scaled_* = (f3 − m)/(M − m), where m and M represent the minimum and maximum *f3* statistic. We ordered ancient genomes in the X-axis according to the time scale. See the abbreviations of the time scale in Figure 4A.

**
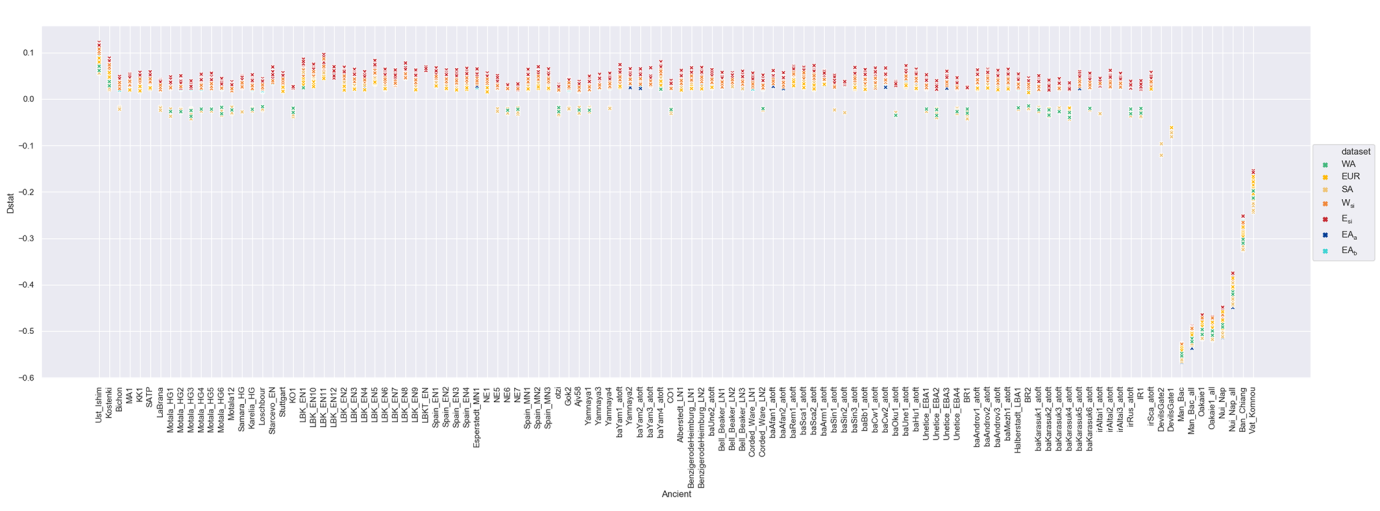
**

**Supplementary Figure 6. *D*-statistics with a form of *D*(Yoruba, Tianyuan, ancient, present-day)**

*D*(Yoruba, Tianyuan, ancient, present-day) test suggests Tianyuan is a putative founder of the EA population by showing significantly positive *D*-stats for the E_si_ population but not for the EA_b_ (none or equal gene flow level). The Tianyuan genome had significantly higher allele sharing with Neolithic than Ion Age Southeast Asian ancient populations compared to present-day EA or Siberian populations and these sharing statistics value decreased over time. It supports the scenario of continuous Tianyuan-derived genetic association with Southeast Asians before and during the Neolithic Age until the Bronze Age.


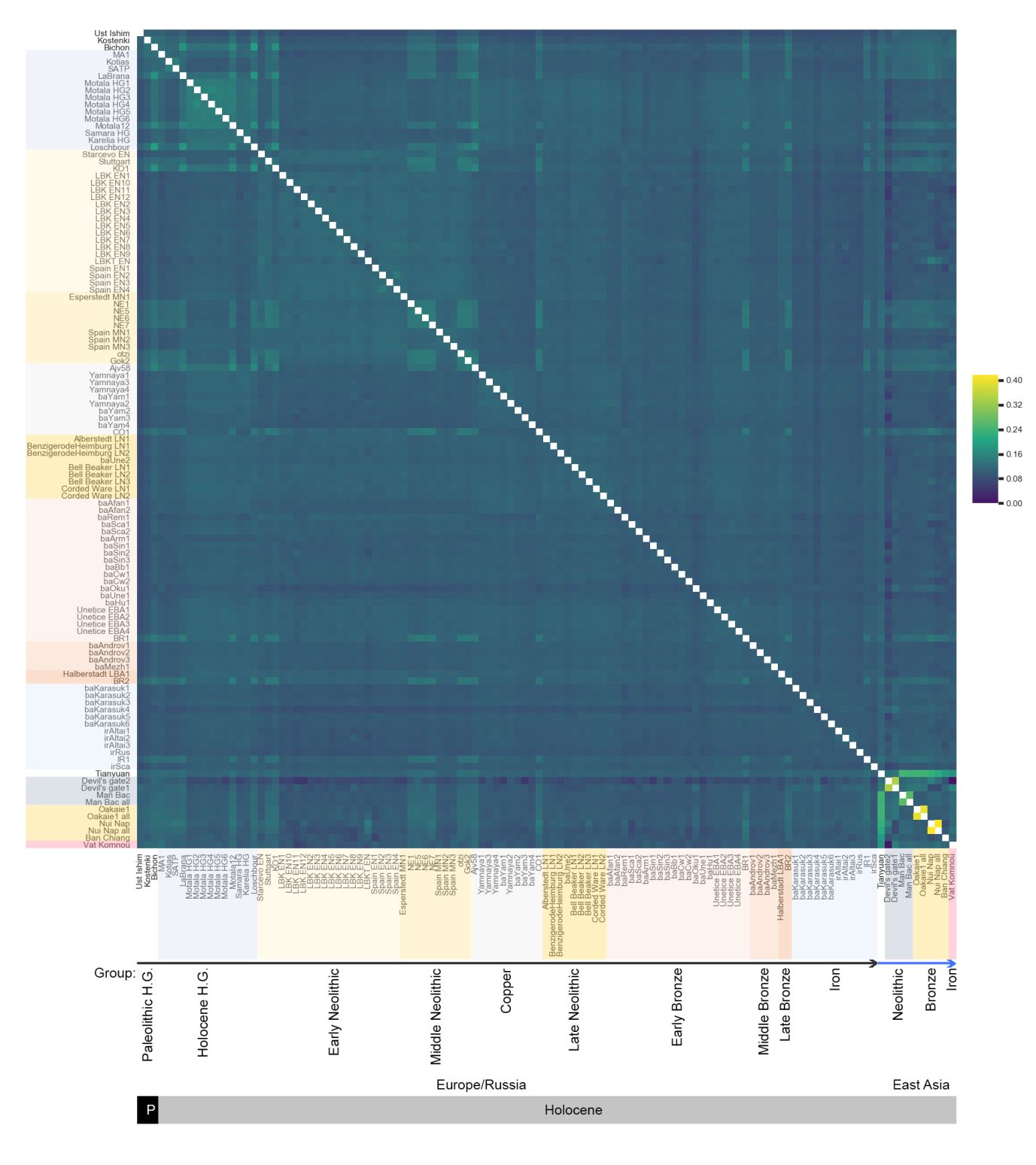


**Supplementary Figure 7. Genetic cluster of the ancient genomes analyzing with outgroup *f3* statistics**

**
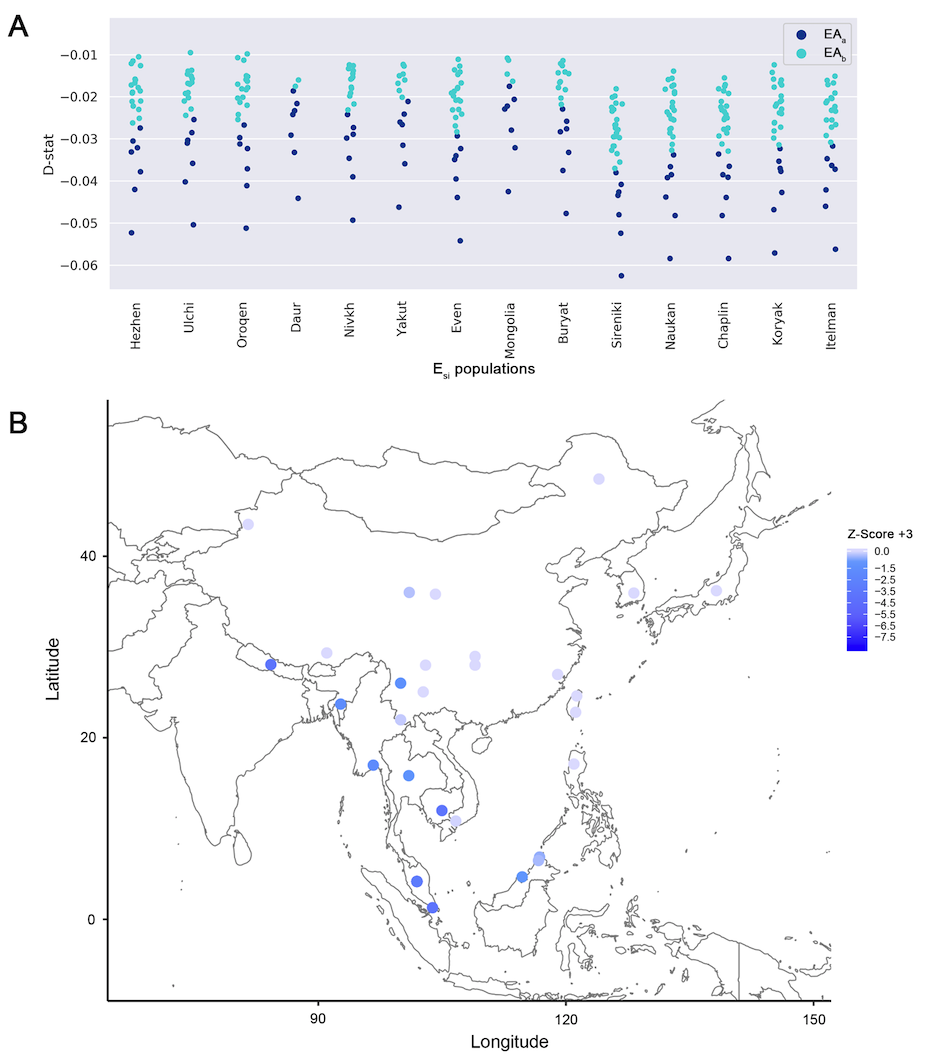
**

**Supplementary Figure 8. *D*-statistics with a form of *D*(Yoruba, Tianyuan; E_si_, EA_a/b_)**

**(A**) The *D*-statistics with a form of *D*(Yoruba, Tianyuan, ancient, present-day) showed that the Tianyuan genome is closely related to E_si_ populations in comparison with EA_a/b_ populations. However, several EA populations (Ami, Atayal, Daur, Hezhen, KOR, Miao, She, Tujia, and Xibo) showed similar levels of divergence from Tianyuan compared to E_si_ populations. (**B**) To represent gene flow and geographical relationships in Tianyuan, we represented the Z-score of *D*(Yoruba, Tianyuan; Yakut, EA_a/b_). For spot colors, “Z-score +3” values are used to represent “0” for individuals that are not statistically significant.

**
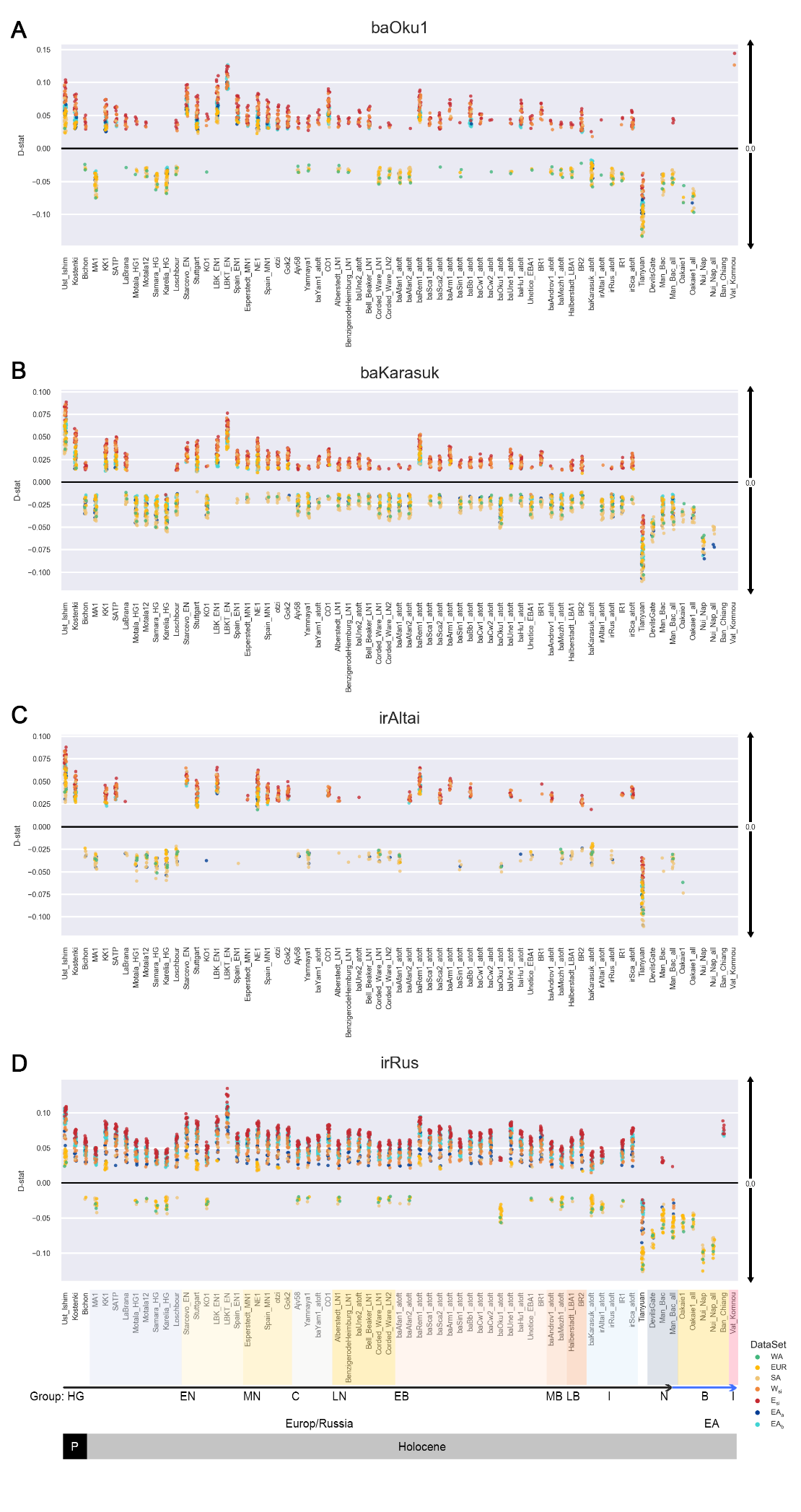
**

**Supplementary Figure 9.** ***D*-statistics with a form of *D*(Yoruba, ancCS; ancient, present-day)**

*D*(Yoruba, ancCS, ancient, present-day) test suggests ancCS genomes are genetically closer to present-day E_si_ populations than European ancients. It also inferred that present-day E_si_ and EA populations and ancSEA are equally close to ancCS, relatively. We represented only the |Z-score| >3 for each *D*-statistics. The positive values represent genetic ancestry to present-day populations and the negative values represent genetic ancestry to ancients at the bottom. Abbreviations for Ancient genome clustering refer to Supplementary Figure 5 and 7.

**
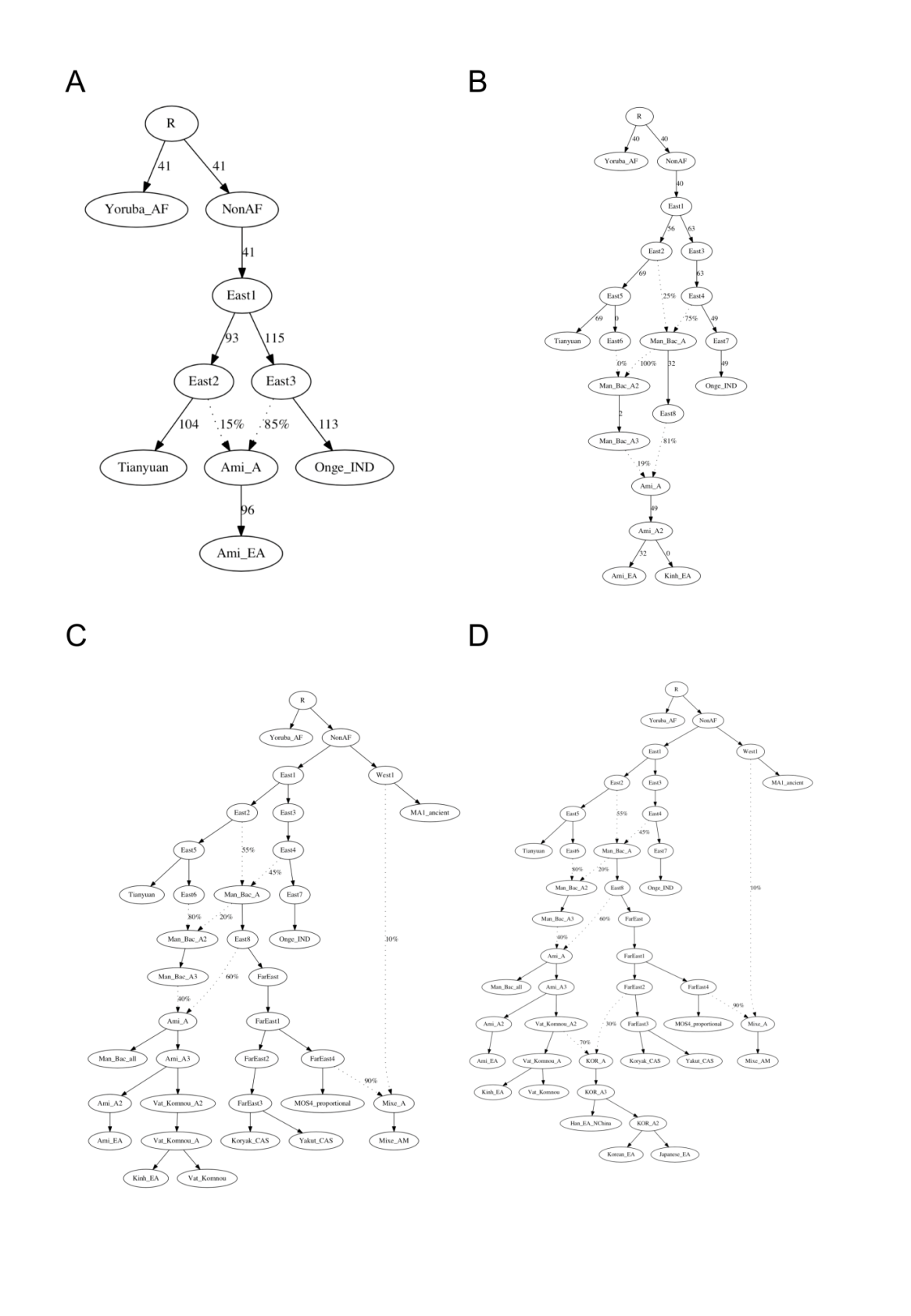
**

**Supplementary Figure 10. Four tested admixture tree models by qpgraph.**

(**A**) Admixture tree model skeleton adapted by previous reports ([Lipson, et al. 2018](#_30j0zll)) (worst-fitting Z=0.044). (**B**) Admixture tree model after adding Kinh from the first model (worst-fitting Z=-3.887). (**C**) after adding Far East samples (Devil’s gate, Ulchi, Koryak, Mixe, and MA1) (worst-fitting Z=3.317). (**D**) Final admixture tree model by adding the Korean, Han, and Japanese populations (worst-fitting Z=-3.686)

**References**

Lipson M, et al. 2018. Ancient genomes document multiple waves of migration in Southeast Asian prehistory. Science 361: 92-95. doi: 10.1126/science.aat3188
